# Endured and prevailed: a phenomenological study of doctors’ first year of clinical practice

**DOI:** 10.1186/s12909-023-04059-w

**Published:** 2023-02-13

**Authors:** Niamh Coakley, Paula O’Leary, Deirdre Bennett

**Affiliations:** 1 Department of Medicine, University College Cork, National University of Ireland, Cork, Ireland; 2 School of Medicine, University College Cork, National University of Ireland, Cork, Ireland; 3 Medical Education Unit, University College Cork, National University of Ireland, Cork, Ireland

**Keywords:** Transition, Clinical practice, Medical graduates, Phenomenology, Clinical learning environment, First year of practice

## Abstract

**Context:**

The challenging nature of the transition from medical student to doctor is highlighted by the associated negative consequences to new doctors’ mental health and wellbeing. Enhanced understanding of the lived experience of recent medical graduates as they move through the stages of transition over the first year of practice can inform interventions to ease the difficulties encountered.

**Methods:**

Using interpretative phenomenological analysis (IPA), a novel approach to this topic, we explored the lived experience of transition from student to doctor over the first year of practice after graduation. Twelve new graduates were purposively recruited. We conducted semi-structured interviews at the end of their first year of practice with respect to their experience over the first year.

**Results:**

The experience of transition was characterised by overlapping temporal stages. Participants’ initial adjustment period was characterised by shock, coping and stabilisation. A phase of development followed, with growth in confidence and a focus on self-care. Adversity was experienced in the form of interprofessional tensions, overwork, isolation and mistreatment. Finally, a period of reflection and rationalisation marked the end of the first year.

**Discussion:**

Following initial anxiety regarding competence and performance, participants’ experience of transition was predominantly influenced by cultural, relational and contextual aspects of clinical practice. Solutions to ease this challenging time include stage-specific transitional interventions, curricular change at both undergraduate and postgraduate levels and a re-evaluation of the clinical learning environment to mitigate the difficulties endured.

**Supplementary Information:**

The online version contains supplementary material available at 10.1186/s12909-023-04059-w.

## Introduction

Although the transition from medical student to doctor has been the focus of much research in medical education, it remains problematic, with persistent reports of a deterioration in doctors’ mental health and wellbeing during this time [1, 2]. The enduring nature of the challenges encountered by medical graduates, across countries and specialties, points to issues common to the transition experience [1, 3–6]. Commencing work has been described as a major change for new graduates, who can lack confidence and feel anxious in their role, with new responsibility and changing relationships with their workplace colleagues [7–11]. Issues relating to support, workload, workplace relationships and role ambiguity contribute to psychological distress in this group [12, 13]. Challenges to new doctors’ professional identity formation or personal growth during the first year of practice include adapting to the medical culture, and issues with confidence, work-life balance, self-care, and interactions with patients and colleagues [14–16].

The transition from medical student to doctor has been referred to, as a ‘rite of passage’, a process over time, involving different phases of, separation (withdrawal or detachment from a previous status or identity), transition (the in-between or ‘liminal stage’), and incorporation (assumption of new status or identity) [17–19]. This resonates with our understanding of the transition as an ongoing, dynamic process of professional discontinuity, with movement from ‘one context and set of interpersonal relationships to another’ [20], encompassing changes in ‘expectancies, tasks or responsibilities’ [21, 22]. The dynamic nature of this transition is mirrored in the variations in mood, empathy, stress and wellbeing reported in new doctors over the course of the first year [23–26], with their wellbeing shown to deteriorate during this time. Longitudinal studies, have described the experience over the first year in terms of a ‘preparedness journey’ with the development of competence and confidence over time [5, 11, 27, 28].

In summary, the current understanding of the first year of practice is of an intense formative experience involving different challenges and stressors with temporal variation in emotion and wellbeing. However the variety of cultural and contextual factors that influence the behaviour of new doctors [29] suggest that the actual experience of the transition as a dynamic transformative experience over time is more complex than is currently understood. We argue that deeper appreciation of this process is critical as a forerunner to intervention.

Research into other workplace transitions can inform our understanding of the process of transition to practice for medical graduates. Nicholson’s theory of work role transitions, describes a cycle in perpetual motion, each stage having different sets of tasks, expectancies, problems and solutions, and comprising of, prior preparation, followed by the initial encounter, a period of adjustment and a stabilisation phase [30]. Regarding transitions in healthcare, extensive work has been carried out on the transition of nursing graduates to professional practice [31, 32]. Kramer described an initial ‘honeymoon stage’ during orientation, characterised by idealism and excitement, followed by ‘reality shock’, where the disparity between expectations and the reality of practice provoked feelings of anxiety, doubt and confusion. After this stage comes ‘recovery’ with reduced anxiety and coping, and finally ‘resolution’ resulting in success or burnout [31]. Informed by Kramer, Duchscher expressed the transition of nursing graduates over the first year in terms of ‘a complex but relatively predictable array of emotional, intellectual, physical, sociocultural and developmental issues that in turn feed a progressive and sequential pattern of personal and professional evolution’ [32, 33].

The majority of qualitative research to date into the transition from medical student to doctor, described here, has been limited to thematic analysis, with other studies employing a grounded theory methodology. Enhanced understanding of the experience of new medical graduates is critical to wellbeing, and would inform interventions to ease the difficulties encountered. An exploration of the lived experience of recent medical graduates over the first year of practice using a phenomenological lens, specifically interpretative phenomenological analysis, has not been carried out. Therefore, we aim to advance the current understanding of the transition by exploring the lived experience of recent medical graduates as they move through the stages of transition over the first year of practice using the contemporary phenomenological approach of interpretative phenomenological analysis. This study is part of a larger project exploring the lived experience of recent graduates as they anticipate their transition to clinical practice and of their experience over the first year of work [34].

## Methodology

This study was carried out within an interpretative paradigm using interpretative phenomenological analysis (IPA) as originally described by Jonathan Smith, of Birkbeck, University of London in 1996 [35]. The theoretical underpinnings of IPA include phenomenology, hermeneutics and idiography. Phenomenology focuses on how individuals make sense of the world and aims to provide insightful accounts of subjective experience [36, 37]. IPA is connected to the hermeneutic tradition, in it’s acknowledgement of the role of the researcher in making sense of the individual’s account of their experience [38]. Smith has described a hermeneutics of empathy in IPA’s attempt to achieve an insider’s perspective, and a hermeneutics of questioning with its interpretive undertaking to analyse and make sense of the experience [35]. IPA involves a ‘double hermeneutic’ in that, the participant provides an account of how they have made sense of their experience, and the researcher, interprets this account, potentially revealing insights beyond the direct assertions of the participant [35]. Finally, IPA involves an idiographic commitment with its focus on a detailed exploration of the particular. An in-depth exploration of each individual case is carried out before analysis of subsequent cases, after which, similarities and differences between cases can be explored [35]. Further detail on the methodology chosen, is provided in the first paper arising from this project, exploring the experience of anticipation of commencing work as a doctor, from the perspective of new medical graduates [34].

### Context

Internship in Ireland is a twelve-month period of pre-registration training following graduation from medical school. This study was carried out in the Southern Intern Training Network, one of six networks nationally with responsibility for this first postgraduate year of training. Interns spend a minimum of three months in General Surgery and three months in General Medicine. The remaining two, three-month, rotations are spent in other specialties such as Paediatrics, Psychiatry, Surgery, and General Practice. Each post involves the newly qualified doctor working within a particular specialty for the duration of that rotation.

Ethical approval for this study was granted by the Clinical Research Ethics Committee of the Cork Teaching Hospitals Ireland. NC, POL and DB are medical doctors with roles in the undergraduate medical programme linked to the Southern Intern Training Network. POL also has a role in the Intern Training Network. NC, who carried out recruitment and interviews, had no oversight of the participants as interns. The interviews were anonymised prior to analysis by the rest of the research team, and pseudonyms were used in the reporting of results. The wellbeing of participants was considered at all stages of the study, including the potential need for support or intervention, however no such need arose.

### Recruitment

Participants were purposively recruited, with respect to gender and graduate entry to medical school or entry directly from high school. All were in their first year of postgraduate practice in the Southern Intern Training Network, working in affiliated hospitals and primary care settings. Contact emails were obtained from the Network administrator and thirtyfive recently qualified doctors were emailed and invited to participate.

### Data collection

Individual semi-structured interviews were carried out with participants as they neared the end of their first year (Interview schedule- appendix 1), with respect to their experience of the first year of practice. Interviews were audio recorded, transcribed verbatim, and anonymised.

### Data analysis

Data was analysed using interpretative phenomenological analysis [35, 39]. The research team, NC, POL and DB met regularly to discuss all aspects of data analysis. Analysis began with immersion and familiarisation with the data by listening to, reading and re reading each interview. Experiential themes were identified (recurrent experiential assertions), which were then grouped into clusters of themes addressing related issues called superordinate themes. Each transcript was analysed fully prior to moving on to the next one. When all data was analysed, integration of themes across the entire dataset was carried out and commonality and divergence were noted. NVivo software was used as a data management tool [40].

### Reflexivity

We acknowledge our role as researchers in the co-construction of knowledge with participants. As faculty members of the medical school aligned with the Southern Intern Training Network, we were aware that as ‘insiders’ we would have preconceptions and beliefs about the research question. We critically reflected on how our prior experience and beliefs about the phenomenon may impact on the research process. While familiarity with the nature and language of the context could be beneficial, we were cognisant that our prior experience would not translate into knowledge of how others might experience the phenomenon. Throughout the research process, the research team continually reflected and discussed on how our attitudes might be informing the research. We supported each other’s reflexivity and kept reflexive diaries throughout the process.

## Results

Twelve participants agreed to take part in these interviews, six males, (two who had prior degrees and four who commenced their medical degrees directly from high school) and six females (two who had prior degrees and four directly from high school).

**Fig. 1:**
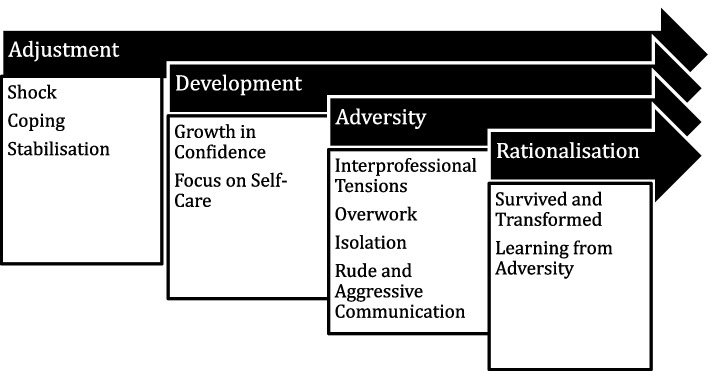
Superordinate themes and their subthemes

We identified superordinate themes of, *adjustment*, *development*, *adversity*, and *rationalisation* (Fig. 1). These themes reflected overlapping temporal phases in the transition experience.

Commencing work provoked an emotional response from participants, and prompted compensatory actions and dependence on others in the workplace. In time, participants became more comfortable in their roles, however, fulfilling the requirements of the job entailed endurance of challenging working conditions, lack of support and difficult workplace interactions. Finally, from their perspective at the end of the year, having prevailed over the challenges encountered, participants reflected on their role, and on their personal evolution.

### Adjustment

This theme was comprised of subthemes of *shock, coping* and *stabilisation*. Many participants experienced negative emotions, including anxiety and self-doubt during the early phase of commencing practice. Support was appreciated, and some dysfunctional coping strategies were employed to fulfil workplace expectations. Participants’ critical gaze at this stage was predominantly inward-looking, focusing on themselves and their performance. As time progressed, their initial fear and anxiety abated as they became more comfortable in their new role.

#### Shock

Words such as *terrifying, overwhelming* and *very stressful* were used by participants to describe their early days as a doctor. New responsibility, role uncertainty and fear of making mistakes contributed to the fear and anxiety experienced by most, to varying degrees in the early days.

*‘The first week was absolutely terrifying and everything was a challenge at that point, from taking blood to cannulas to prescribing to being called to a sick patient and having to assess them, everything was hard at the start’* ***Jane***

Most struggled initially with prioritisation of workload and the unique pressure of the bleep system, especially when they were on call at night, which was stressful and sometimes overwhelming.

*Responding to calls on your own and being overwhelmed by bleeps and not being able to triage things effectively is really and can be really quite overwhelming* ***Gerard***

Not all early work experiences were difficult. Andrew and Brian described a gentler transition which did not include these negative emotions, however both expressed frustration and disappointment that their first job did not require them to assume responsibility.

*So, very little sort of using your brain, but it was an ok one to start off on for maybe the first 6 weeks until you realise this is actually not medicine* ***Brian***

#### Coping

Coping strategies in the early weeks included working longer hours, missing breaks to fulfil their obligations and depending on others in the workplace for support and guidance.

*And again, coming in earlier and getting it done so you wouldn’t be delaying the surgeons* ***Claire***

*I used to like not eat during the day on the day job because I would be like, I had so much to do.* ***Mark***

Their nursing colleagues were crucial sources of early support. Participants felt reliant and dependent on them, willingly accepting their direction without question.

*I think at the beginning … they are your best allies and I think you lack confidence in yourself so you do whatever they say. If they say jump, you’re just, ok how high* ***Faye***

All, also acknowledged the benefit of integration, camaraderie and support that derived from a team structure and the solidarity of other interns was greatly valued.

I had a fellow intern so the two of us used to go around together so having him made everything so much easier … We had 4 *SHOs … they were great as well ... So it was good, it was a nice team, the structure kind of made it. ***Deirdre*** *senior house officers

#### Stabilisation

The initial negative feelings of shock abated with time. This marked the transition to the next phase of the experience for participants.

*I was afraid of making mistakes, that I was going to hurt someone, but then that eases off … gradually you get a little more confident as you go along* ***Jane***

### Development

This superordinate theme comprised of subthemes of *growth in confidence* and *focus on self-care*. After the initial adjustment period, participants continued to evolve, developing increasing confidence and competence. This resulted in a change in some of their earlier attitudes and perspectives.

#### Growth in confidence

As the year progressed, participants became more competent and confident, characterised by their assumption of increased responsibility, making clinical decisions and prioritising their workload.

*I think for me that was the active point of transition where I went from just blindly following orders … to actually employing my own clinical judgement* ***Gerard***

Gradually, their requirement for support lessened and their attitudes towards requesting support also changed.

*I would have looked at asking for help a bit differently early on .. felt a bit bad about having to ask .. now I realise I have brought this to where I can bring it .. and part of your job almost is to get further help* ***Hannah***

#### Focus on self-care

In contrast to the initial days of transition, participants recognised the importance of self-care, and of maintaining a work life balance.

*On long days .. when you are run off your feet … it was about teaching myself that I needed to stop … to take a break even if it was only 20 minutes or something .. because I would work better after that* ***Jane***

*Keeping up hobbies and things outside work … that has been really important to me.. seeing friends and talking with them … It is important to take your mind off things* ***Gerard***

### Adversity

This superordinate theme comprised of subthemes of *interprofessional tensions, overwork, isolation*, and *rude and aggressive communication*. Following participants’ initial adjustment to the clinical environment, their relationships with their nursing colleagues began to change. During this stage, their main focus shifted from their own abilities or shortcomings, to exogenous workplace stressors. All described experiences of feeling overworked, unsupported, or subject to difficult workplace interactions with more senior doctors, for example when requesting consults or investigations, which they accepted and endured.

#### Interprofessional tensions

As their first year of practice progressed, rather than simply complying with all the demands placed on them, participants became more assertive. This sometimes resulted in tensions developing with their nursing colleagues.

*The nurses are more of a challenge now .. at the beginning .. whatever they said, I did, because I didn’t know, whereas now you are questioning them more and it is a little bit more of a challenge.* ***Deirdre***

Faye addressed what she perceived as the strong division between interns and their nursing colleagues, relating this, in part, to their different working practices.

*There is definitely quite a strong division between doctors and nurses .. they* [nurses] *have no consideration, they are on a ward and they look after their room and they know their patients well. They have no kind of notion … that you could have patients on every ward of the hospital and you are up and down …. they could bleep you and say I have such and such daughter here she wants to meet you and like I am ...* [busy with a sick patient] *.. and they won’t care and then they will document it 'wouldn’t speak to daughter'.* ***Faye***

Many participants were critical of how their nursing colleagues used the bleep system, including the constant nature of the calls, their tone which was often perceived as pressurising and demanding, and their numerous non-urgent requests, especially on call when participants were often very busy.

They have just decided ... they need a repeat* potassium at 2 in the morning .. there is no point arguing with them because they are just going to keep bleeping you. Irene*blood test

Some participants also struggled with what they perceived as a devolving of responsibility for a patient to them by the nurses, while others felt undermined when nurses would contact senior doctors if they did not respond quickly or appropriately enough.

*They’re all about defending themselves and all about, ‘bleeped doctor up, never came’ you know, there will be no explanation from the doctor documented afterwards, just, ‘never came’ you know* ***Faye***

*And I told the nurses, ‘she’s fine, you don’t need to give her fluids, she’s sleeping, and she’s fine’…. What annoyed me was she went over my head and rang the SHO.* ***Irene***

Gerard and Jane however, contended that nurses are generally more experienced and are therefore entitled to question interns, or seek higher authority, and, that jobs, perceived as trivial by many interns, could be valuable learning experiences and are also important to the patient.

*That is just part of being an intern I think. You are the first port of call and you are the first person to decide is this trivial or is it not… it is only by seeing those as well that you learn what actually is trivial and what is not…. It is important to the patients as well ... if they are worried about something .. it is good to reassure them and things.* ***Jane***

#### Overwork

Most participants had experiences where they felt overwhelmed due to long working hours, intense workloads, and lack of breaks, especially on call. The resulting consequences to their health and wellbeing, included extreme fatigue, irritability, emotional lability and diminished empathy. Participants accepted and complied with the all workplace demands placed on them.

*It was so busy. It was horrible, it was just constantly, my bleep ...…. But that was very overwhelming ... at the end of the shift I felt so emotionally drained I felt that I was going to cry, not from just being upset but from the emotional exhaustion of it you know?* ***Irene***

*When you are tired sometimes it is difficult to muster empathy for patients and that’s meant to be the core of the job you know ... so you feel like that's been chipped away a little bit.* ***Brian***

Faye felt there were occasions when her performance was impaired, potentially compromising patient safety.

*You're hypoglycaemic. You’re overloaded with work. You’re completely not performing to your best and you slow down and then your job ends up longer because you are doing it slower so it is horrendous like ... and you are not even thinking straight. I have been on call where I have put my own date of birth on blood bottles because I cannot even see or think* ***Faye***

#### Isolation

Although in general, participants felt supported, most admitted to occasions when they felt isolated and alone. Support could be variable depending on specialty, individual asked, or time of the day.

*With surgery, you’re thrown in the deep end, because … all the team goes off the theatre for the day, and unless there is something like a massive, massive emergency, you don’t want to go up and interrupt a theatre.* ***Noel***

I think it is lonely, you are on your own, depending on what regs* you are on with, some of them are very helpful .. others a little slower... ***Deirdre ***(support seeking on call) *registrar or senior resident

Most participants recounted episodes where they had difficulty accessing support in spite of requesting it, which caused them great distress.

*There was one night that I think will always haunt me, … we had a really sick patient…and I rang the surgical reg .... [*she said*], I’ll be in A&E for another hour .. you end up pleading like, please,…. and like at 2 o’clock in the morning you’re like, can somebody else just come .. I remember thinking that night, god it’s just me... I remember feeling very unsupported, …. I kind of cried after that, …thinking, god, is this what it’s going to be like every night?* ***Claire***

*Panic and stress initially and then quite frustrated because .. it is not fair on the patient, they shouldn’t be left in a situation where they are sick and I don’t know what to do, and the person who should know what to do isn’t going to come and see them. Frustrated for myself as well because you are told in medical school oh don’t worry you won’t ever be left in a situation where you'll had to deal with something* ***Jane***

#### Rude and aggressive communication

Participants were vulnerable to difficult interactions with senior doctors when they had to contact other teams to request consults or investigations. Participants accepted, rather than challenged, any negative or hostile reactions.

*I remember ringing for consults,.. sometimes you feel afraid, because you wouldn’t know what form you’d find the other person on the phone. ****Claire***

The interactions could be quite unpleasant, distressing and belittling.

‘I remember contacting an anaesthetist ... I was told to contact him and he was going ‘why is an intern contacting me? Your Reg needs to contact me’ and *bang’ ***Deirdre*** (* he hung up the phone)

*One of the interns was brought to tears by a Radiologist and was furious with herself and didn’t do it until after she had left but it was just unnecessary really … and you know interns generally get the brunt of that kind of thing I suppose which is unfortunate.* ***Mark***

Participants described how they accepted mistreatment directed towards them, with Noel perceiving organisational tolerance towards it.

*‘You just learn to live with it’ ****Jane***

*‘They do this sort of thing all the time, they get away with it, it’s never really challenged properly’* ***Noel***

### Rationalisation

This superordinate theme was comprised of subthemes of *survived and transformed,* and *learning from adversity.* This final phase is characterised by a retrospective positive evaluation of the transition, an acknowledgement of the learning opportunities in the challenging aspects of the year and a recognition of personal evolution over the course of the year.

#### Survived and transformed

From their perspective at the end of the year, the most commonly held overall impression was that it was an enjoyable experience, and that they had prevailed over the challenges encountered.

*Yeah I enjoyed it, yeah I liked it and survived it* ***Mark***

Reflecting on the early transition period, initial sources of fear and apprehension seemed much less significant. Participants recognised how they changed over the course of the year.

*The things that worried me back then, now seem a bit silly. Just literally I was so ridiculously stressed about putting in a line, which now I can do in my sleep, so that’s great.* ***Irene***

*It has been a massive transition, I don’t think I would recognise myself if I saw myself approaching a patient back in July now* ***Gerard***

#### Learning through adversity

Many participants acknowledged the role played by the more challenging aspects of the job in their evolution as more resilient, competent practitioners. Intense workloads, acutely ill patients, and working out of hours, although often stressful and anxiety provoking at the time, were now evaluated as important and valued learning opportunities.

*The pace was so much faster, it was a little bit overwhelming at times, .. but I actually think I prefer it like that, because you get more efficient with dealing with things, and learning to do things quickly* ***Irene***

*So it was very much learning on your feet but as terrifying as it is at the time it is nearly the best way to learn.* ***Jane***

## Discussion

### Principal findings

Our analysis identified overlapping temporal stages, as students made the transition to doctor over the first year post graduation. The early transition period was characterised by a high degree of anxiety regarding competence and performance. Support eased the experience, and some dysfunctional strategies, such as skipping meals and breaks, were employed to fulfil the requirements of the role. While the necessary skills and competencies were significant in participants’ early transition experience, beyond this, cultural, relational and contextual aspects of clinical practice were more influential in participants’ experience of the transition. From their perspective at the end of the year, challenges were minimised and reframed as learning experiences.

### Initial experience

Fear and apprehension were prominent early emotions in this study, resonating with existing research on medical graduates’ transition to practice [8, 9, 11]. Organisational socialisation theory describes the early experience of a newcomer to an organisation characterised by sense making and coping, and is often termed ‘reality shock’ or ‘transition shock’. Factors that contribute to this are, a disparity between job expectations and the reality encountered (surprise), and the degree of change (the objective difference between previous and new roles), and contrast (subjective difference as perceived by the individual) encountered [41]. The shock described in this study reflects the degree of surprise, change and contrast experienced by participants in their new roles compared with their previous roles as medical students [7, 42].

Participants’ expectations of their performance did not include any allowances for the early transition period. High personal expectations, and concerns regarding competency increase the stress of the transition [7, 22]. The dysfunctional coping strategies employed to compensate for perceived inefficiency, reflect the pressure on novice doctors to ‘keep on top of things’ [43], and perhaps the common assumption in medicine of doctors as ‘supernaturally resilient’ [44]. A more positive coping strategy was participants’ support-seeking from workplace relationships. Access to insiders can provide the context-specific experiences that newcomers lack, helping them to make sense of their new reality and facilitating enhanced understanding of their new environment [45]. Support and team integration [46, 47], positively impacts on feelings of preparedness for transition [48], improves work related wellbeing [23, 25], creates a positive working environment and reduces the stress around transition as seen in this study [8, 10, 23, 25, 27, 49].

### Inter-professional collaboration

The early reliance on nursing colleagues by doctors commencing clinical work, is not a novel finding [23, 47]. Nurses play an important but often unacknowledged role in the socialisation of new doctors [50, 51]. Participants displayed an initial vulnerability and willingness to accept their direction without question, as they acclimatised to their new roles. This resonates with Burford et als’ ‘pragmatic hierarchy’ where the experience and expertise of nurses carries an authority to which participants were initially happy to defer [50].

Time and experience in the clinical environment diminished this initial collaborative practice. As participants began to critically appraise the requirements of their role, tensions became apparent. A number of cultural and organisational barriers to collaborative practice have been described [52–54]. Doctors and nurses appear to identify primarily with their own teams and only secondarily with the interprofessional team, illustrating the ‘tribal behaviours associated with the discrete cultures of the different professions’ [52, 55]. Their individual work practices differ substantially, while nurses are generally ward based as part of a team, with a set number of patients to care for, first year doctors work as ‘inter-professional isolates’, caring for a variable number of patients, on different wards, often with limited senior support and are only transiently present prior to moving to their next job [52]. Some participants felt undermined by their nursing colleagues at times. As the ‘emergent identity’ of new doctors can be affirmed or disaffirmed by others in the workplace, friction can result if other healthcare professionals either do not see them as experienced enough to claim the authority they desire, or if their expectations of the novice doctors are too high [56].

### Mistreatment

This study further highlights the pervasive issue of mistreatment of residents [57–61]. Experiencing or witnessing this behaviour negatively impacts on health and wellbeing, with reports of psychological distress, post-traumatic stress disorder, professional demotivation, burnout, dissatisfaction with the job and with training, and thoughts of leaving [57, 59, 60, 62–67]. Patient care is also adversely affected, with an increase in errors, disruption to collegiality and communication and diminished teamwork and morale [57, 59, 60, 62–69]. Mistreatment often remains unreported by residents [59–61, 64, 70–72]. Senior doctors from other departments were cited as perpetrators of mistreatment towards participants in this study, which has been reported previously [62, 73]. This reluctance to challenge seniors may indicate fear, or vulnerability in the medical hierarchy, or residents’ socialisation into the culture of medicine as transmitted by the hidden curriculum, with the acceptance of mistreatment as a way of signifying their worthiness for the profession [43, 64, 74–76]. Community of practice theory describes learners actively engaging with, and acquiring the identity associated with the community, who exert ‘a compelling social influence on its members’ [77–79]. Residents may assimilate the negative behaviours they are exposed to via the hidden curriculum and themselves become perpetrators, continuing a legacy of abusive behaviours [64, 80]. It is noteworthy that participants in this study appraised their job positively in retrospect. Earlier difficulties were downplayed to some extent and they evaluated the more challenging aspects of the year as valuable learning experiences. This may imply that they have acquired important experiences in order to work as doctors in the future, however it may also indicate an element of denial of difficulties encountered [23, 47, 81]. It has been suggested that doctors tend to trivialise or normalise their experiences of stress as a form of impression management to demonstrate professionalism [82] This attitude has the potential to maintain negative workplace culture for future residents [83].

## Implications for practice

This study reveals overlapping temporal stages of the transition experience for new doctors. The themes that have been identified will inform stage-appropriate interventions to ease the transition. To mitigate the shock experienced in the initial stage of the transition, an undergraduate practice-based pedagogy such as Experience Based Learning (ExBL) would facilitate supported participation in clinical practice, and empower students to acquire intellectual, practical and affective capabilities gradually over time [84]. Anxiety and self-doubt characterised the early phase and support eased participants’ experience. Acknowledgement of the early transition period should include organisational expectation of initial underperformance, with increased early support and supervision [11, 85]. Consideration could be given to a period of overlap between incoming and outgoing interns, and faculty development to enable senior colleagues to support new doctors. Participants identified their nursing colleagues as key sources of early support. The contribution of nurses to new doctor socialisation, should be formally recognised and interventions designed to facilitate nurses to provide appropriate support [51, 85].

To mitigate the interprofessional tensions evident in this study, undergraduate and postgraduate education should include interdisciplinary involvement to promote role understanding, mutual respect, trust and collaboration and any barriers to collaboration identified and addressed [52, 86–89].

Focused interventions to tackle mistreatment of residents should target organisational, team and individual levels commencing with a zero tolerance policy of unprofessional behaviour, transparently monitored and enforced [67]. Education aimed at all levels in the organisation should include shared mental models as to what qualifies as mistreatment, facilitating the recognition and reporting of any problematic behaviour [64]. Residents need to feel psychologically safe in the clinical environment, to speak up or ask for assistance without fear of a hostile reaction [90]. Healthcare organisations must address issues within their own cultures and ensure that positive and supportive workplace relationships are fostered [91], including the education of senior residents who are responsible for supervising more junior doctors [92].

A culture that promotes student and resident wellness and resilience in undergraduate and postgraduate curricula should be a priority, with positive leadership, targeted interventions and the provision of resources promoting resilience and self-care. Interventions could include resident wellness programs, coaching, mentorship, and mindfulness [93, 94]. However, interventions focusing on the individual are only one aspect of promoting wellness. The work environment of doctors in their first year of practice should be re-evaluated. Residents should not be overburdened by high workload or long working hours, both of which were identified as issues by participants. Duty hour restrictions have been introduced by many countries to regulate working hours, however concerns have been raised regarding a resultant increase in work intensity due to pressure to complete workload with less time [23, 95]. A potential solution may be the introduction of an optimal patient caseload per resident, depending on specialty or patient complexity [96, 97] or strategies designed to minimise interruptions or the geographical distances residents cover [97].

## Strengths and limitations

The use of interpretative phenomenological analysis, which is acknowledged as being a particularly effective methodology to explore experiences of existential importance to individuals [35], has resulted in a nuanced, in-depth understanding of the experience of transition from student to doctor in this study.

There are some potential limitations to consider. All participants were recruited from one intern training network. This has potential consequences for wider applicability, however, it is our intention that the reader may evaluate the transferability of conclusions, drawn in the context of this study, to other settings. To facilitate this a rich detailed description has been provided of the context, methodology and all research processes, and findings are discussed with reference to the extant relevant literature [98]. NC carried out all interviews. As a member of the faculty of the medical school aligned to the intern training network, she was known to many of the participants. Although she had no involvement in the intern programme, it is possible that this may have resulted in participants not being fully open in their accounts. Furthermore, her position as an ‘insider’ with prior experience of the subject of the inquiry, may have resulted in her failure to probe elements of participants’ experience sufficiently. It is possible therefore that aspects of the experience remain underexplored.

## Conclusions

This study adds to the existing knowledge on the transition to clinical practice by using a phenomenological lens to provide an in-depth exploration of the experience of transition from student to doctor over the first year of practice. Overlapping stages in the transition experience were revealed, informing stage appropriate interventions to ease the difficulties encountered. It also highlights a suboptimal clinical learning environment with issues with workload, supervision, mistreatment by senior doctors, and both the positive contribution of nurses to doctors’ early socialisation and the diminution of inter-professional collaboration with time.

## Supplementary Information

**Additional file 1.**

## Data Availability

The data analysed in this study are available from the corresponding author on reasonable request, subject to ethical approval.
