# Supplementary material for: Endured and prevailed: a phenomenological study of doctors’ first year of clinical practice
Source: BMC Med Educ. 2023 Feb 13;23:109. doi: 10.1186/s12909-023-04059-w (PMC9923928; doi:10.1186/s12909-023-04059-w)
Supplement: Supplementary file 1 — Additional file 1. [file 12909_2023_4059_MOESM1_ESM.docx]

**Appendix 1**

**Interview Schedule**

Transition to Internship Study

**To be conducted** **towards the end of internship**

Looking back on your internship what were the biggest challenges you faced initially?

How did these evolve over time?

How did the experience compare to your expectations?

What kind of work did you spend your time doing?

How were your relationships with other doctors and allied healthcare professionals?

What level of support did you receive?

How did you cope with working hours?

What kind of challenges did you encounter?

How could the undergraduate curriculum have prepared you better?
